# Neurodisability with Severe Restriction of Mobility Is Associated with Reduced Serum Creatinine Values

**DOI:** 10.1155/2019/3598123

**Published:** 2019-06-16

**Authors:** Chun Lim, Marianne Phillips, Liam Watson, Michael Eisenhut

**Affiliations:** Luton&Dunstable University Hospital NHS Foundation Trust, Lewsey Road, Luton, LU4ODZ, UK

## Abstract

**Introduction:**

We investigated whether reduced mobility is associated with a reduction in serum creatinine in otherwise well children with neurodisability.

**Materials and Methods:**

This was a record based retrospective study and creatinine levels of children in groups with Gross Motor Function Classification System (GMFCS) category 1, 2, or 3 (group I); GMFCS 4 or 5 (group II); and age matched controls (group III) were compared.

**Results:**

Creatinine values were significantly different (p=0.002) between patients with neurodisability (mean 32.0 (SD 9.3), n=88) compared to patients without (mean 36.5 SD (9.8), n=88). There was no significant difference in creatinine levels (p=0.684) between group I (mean 35.6 (9.1), n=23) and group III (mean 36.5 (9.8), n=88). A significantly lower creatinine level (p<0.001) was found in group II (mean 30.7 (9.1), n=65) compared to group III and group I compared to group II (p=0.034). Creatinine levels were with a mean (SD) of 25.7 (4.1) micromol/l significantly lower in patients with GMFCS 4 or 5 who died (n=4) compared to survivors (31.1 (3.6)), (p=0.04, n=61).

**Conclusions:**

Children with neurodisability with severe mobility restriction had a significantly lower serum creatinine compared to controls with less severe or no neurodisability. Death in severe neurodisability may be associated with lower creatinine levels.

## 1. Introduction

Creatinine is produced from the conversion of creatine and creatine phosphate. About 95% of this compound is found in muscle. The concentration of creatinine in peripheral blood is therefore dependent on muscle mass [1]. Low serum creatinine (SCr) levels have therefore been associated with low muscle mass due to female gender, more advanced age, chronic illness, malnutrition, low protein diet, advanced liver disease, fluid overload, and augmented renal clearance states like pregnancy or a systemic inflammatory response syndrome in critical illness [2]. In this case control study we are to our knowledge the first who compare urea and creatinine levels in children with neurodisability with age matched controls without neurodisability to investigate whether well children with neurodisability have different creatinine levels.

## 2. Methods

### 2.1. Study Design

The study was designed as a case record based retrospective case control study.

### 2.2. Ethical Approval and Consent

The project did not require ethical approval or consent because it fulfilled the criteria for clinical audit set by the National Research Ethics Service of the National Patient Safety Agency of the United Kingdom including design and conduct to produce information to inform delivery of best care. For this type of study formal consent is not required [3].

#### 2.2.1. Inclusion Criteria

Inclusion criteria: It included all children (>1 and <16 years of age) recorded on a paediatric physiotherapy database with neurodisability. This is a comprehensive database for such children within the area of Luton town, United Kingdom, and surrounding areas because most patients from this area are referred for physiotherapy assessment to the hospital physiotherapy department.

#### 2.2.2. Exclusion Criteria

Excluded were patients younger than one year of age, on nephrotoxic drugs, with known renal disease and those who had no blood sample taken or only had blood tests done when potentially dehydrated at the time of blood sampling.

#### 2.2.3. Laboratory Methods

Creatinine measurements were performed in the Laboratories for Clinical Biochemistry of the Luton&Dunstable University Hospital NHS Foundation Trust by the Jaffe method [4].

#### 2.2.4. Data Analysis

Patients with neurodisability were compared to age matched patients without neurodisability with regard to urea and creatinine levels, gender, and weight. In addition patients in the following groups were compared: group I: Gross Motor Function Classification System (GMFCS, for definition see below) category 1, 2, or 3; group II: GMFCS 4 or 5; and group III: age matched controls without neurodisability. To investigate the influence of the constant nutritional and fluid support given by nasogastric, gastrostomy, or jejunostomy feeds on creatinine levels we compared creatinine levels between patients with and without nutritional support by tube feeding. We also compared creatinine levels in II between patients who had died by the time of data collection and patients who were still alive.

### 2.3. Data Processing

Data for patients with neurodisability were identified and processed after transfer from clinical databases (Sunquest. ICE®) and Evolve, (Kainos, Ltd) ) onto Microsoft Excel 2010 files in anonymized form on password protected computers on the premises of the physiotherapy and paediatric departments of the Luton&Dunstable University Hospital NHS Foundation Trust in Luton, United Kingdom, where this project was registered as an audit project. Age matched controls without neurodisability were identified from hand written phlebotomy records of the children's outpatient department of the same hospital and their data accessed and processed on the above mentioned data bases. Blood tests results were taken from the first result obtained electively in an outpatient or inpatient setting when the patient was well but electively assessed to screen for chronic renal involvement or electrolyte imbalance. We extracted data on age, gender, comorbidity, urea and creatinine levels, and the degree of neurodisability as categorized by the Gross Motor Function Classification System (GMFCS) [5, 6]: GMFCS Level 1: children walk at home, at school, outdoors and in the community. They can climb stairs without the use of a railing. Children perform gross motor skills such as running and jumping, but speed, balance, and coordination are limited. GMFCS Level 2: children walk in most settings and climb stairs holding onto a railing. They may experience difficulty walking long distances and balancing on uneven terrain, inclines, in crowded areas or confined spaces.

Children may walk with physical assistance, a handheld mobility device, or using wheeled mobility over long distances. Children have only minimal ability to perform gross motor skills such as running and jumping. GMFCS Level 3: children walk using a handheld mobility device in most indoor settings. They may climb stairs holding onto a railing with supervision or assistance. Children use wheeled mobility when traveling long distances and may self-propel for shorter distances. GMFCS Level 4: children use methods of mobility that require physical assistance or powered mobility in most settings. They may walk for short distances at home with physical assistance or use powered mobility or a body support walker when positioned. At school, outdoors, and in the community children are transported in a manual wheelchair or use powered mobility. GMFCS Level 5: children are transported in a manual wheelchair in all settings. Children are limited in their ability to maintain antigravity head and trunk postures and control leg and arm movements.

### 2.4. Statistical Methods

Age, urea, and creatinine levels were compared between groups with (cases) and without neurodisability (controls) using t-test for independent variables. We used a two-tailed t-test for all analyses except for the comparison of creatinine levels in patients with GMFCS 4 or 5 who died to patients who survived where we used a one tailed t-test because based on previous study results [2] we were only interested in confirmation of the previous findings of a lower creatinine being associated with death. For comparison of creatinine levels between groups with different degrees of neurodisability we used analysis of variance (ANOVA) with least significant difference (LSD) as post hoc test for significant difference between groups. To determine whether significant differences between cases and controls were related to the differences in creatinine between groups we used multiple linear regression analysis inserting creatinine as dependent and the presence of neurodisability and weight as independent variables.

The statistical software IBM SPSS version 25 was used for the corresponding statistical analysis. For comparison of gender proportions Epi-Info version 6.04b (CDC Atlanta, USA) was used. A p-value of <0.05 was taken as indicating statistically significant difference.

## 3. Results

176 children were included: 23 children in group I, 65 in group II, and 88 in group III. Children in group I and group II had as cause of neurodisability cerebral palsy in 49, global developmental delay with or without brain malformations of unknown cause in 24, and identified genetic disorders in 13, SSPE or stroke in one each. Controls had underlying haematological disorders (thalassemia, sickle cell disease, or iron deficiency anemia) in 12, gastroenterological conditions without diarrhoea in 16, 10 each with respiratory or dermatological conditions, endocrinological conditions in 5, surgical conditions or viral illness in 4 each, and others in 27. Age at time of phlebotomy and gender were not different between groups. The majority of patients (69%) had their blood sample taken in the outpatient setting without difference between the groups. Creatinine values were significantly lower in patients with neurodisability compared to patients without (see Table 1). On multiple comparison testing there was a significant difference in creatinine levels between groups I, II, and III (ANOVA p=0.001). On post hoc analysis there was no significant difference in creatinine levels (p=0.684) between group I (mean 35.6 SD 9.1) and group III (mean 36.5 SD 9.8). A significantly lower creatinine level (p<0.001) was found in group II (mean 30.7 SD 9.1) compared to group III and group I compared to group II (p=0.034). There was no difference in creatinine levels where these data were available (n=80), between patients with neurodisability receiving tube feeding (n=33) with 31.2 (9.7) and patients not receiving tube feeding (n=47) with 33.5 (9.1) (p=0.284). Weight was significantly lower in children with neurodisability. We therefore performed a multiple linear regression analysis with weight and neurodisability status as independent and creatinine levels as dependent variables to check whether neurodisability influences creatinine levels independently of weight. The result was that with a t=2.7 at a beta of 0.18 neurodisability remained a significant independent predictor of creatinine levels (p=0.007).

**Figure 1:**
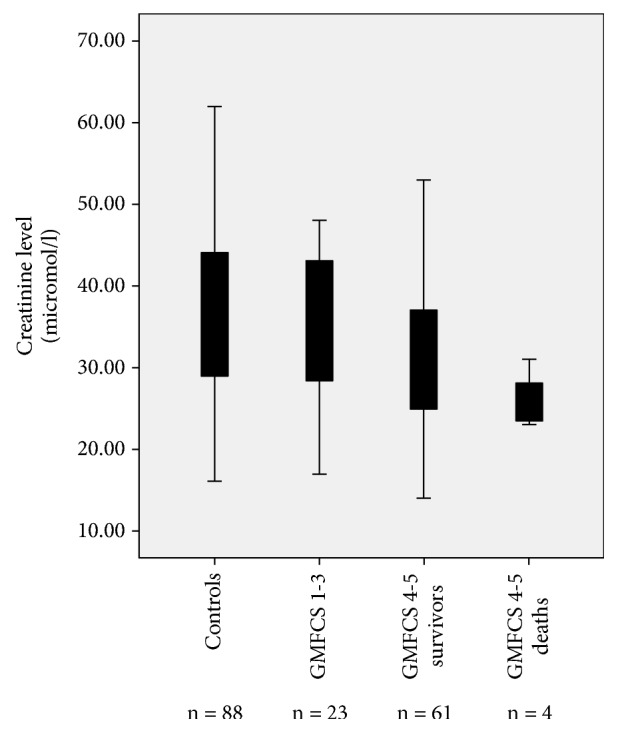
Boxplot of creatinine levels in groups of children with neurodisability with Gross Motor Function Classification System (GMFCS) 1 to 3, GMFCS 4 or 5 who survived, GMFCS 4 or 5 who died, and controls without neurodisability. Lower and higher end of the whiskers represent minimum and maximum and lower and higher margin of the box first and third quartiles, respectively.

In patients with GMFCS 4 and 5, where all deaths occurred, creatinine levels were with a mean (SD) of 25.7 (4.1) micromol/l significantly lower in patients who had died (n=4) compared to patients who had survived (n=61) by the time of data analysis who had a mean creatinine of 31.1 (9.3) (p=0.047). Death had occurred at a mean of 3.9 years (range 1.6 to 8.5) after the creatinine level was done. For a graphic depiction of creatinine levels in the different groups investigated see Figure 1.

## 4. Discussion

The results of our study demonstrate for the first time that children with neurodisability at a level of GMFCS 4 or 5 have lower serum creatinine levels compared to children without neurodisability of the same age and gender distribution. Urea levels were not significantly different between the groups indicating that nutritional protein load or hydration status was not responsible for this difference. The lower creatinine levels in children with neurodisability are therefore likely a reflection of inactivity of muscles leading to muscle wasting and a subsequent reduction in the muscle-based creatinine production. Muscle wasting may have also accounted for the lower weight found in children with neurodisability compared to controls.

It is essential to define creatinine levels for children with neurodisability because people with neurodisability are at increased risk of dehydration from insufficient fluid input due to the reduced ability to communicate particularly at the more severe end of the spectrum of mobility restriction. Lower base line creatinine levels may, if not taken into account, lead to delayed recognition of dehydration if assessed by renal function tests or dehydration induced renal impairment or indeed any other form of renal impairment.

The finding of a lower creatinine levels in children who died with mobility restriction requiring physical assistance or powered mobility in most or all settings (GMFCS 4 or 5) was expected from the finding of an association of reduced survival rates in patients with low admission serum creatinine in the intensive care unit setting. This has been attributed to malnutrition associated low muscle mass and at the same time associated with secondary immunodeficiency and the more intense systemic inflammatory response causing increased renal filtration in more critically ill patients and thus lower SCr levels [2]. To overcome the problem of the influence of muscle wasting, particularly in patients with chronic kidney disease, on creatinine levels, when an attempt is made to predict the glomerular filtration rate from a single measurement of serum creatinine, equations including the capacity for creatinine production from lean muscle are desirable. Since muscle mass and body weight are correlated, equations that incorporate body weight might provide better estimates of GFR. This has to take into account that in children with severe neurodisability muscle may have been replaced by fat tissue and there may in addition be an excess of such tissue because of immobility associated obesity. Macdonald et al. developed a GFR prediction equation that includes bioimpedance measurement to account for skeletal muscle mass and Taylor et al. developed an equation that includes lean body mass measured with dual-energy X-ray absorptiometry but both methods are not available in routine clinical practice [7–9]. What could be made available for routine clinical practice is a method suggested by George J. Schwartz [10], which is the measurement of cystatin C.

To quote his description of this method and its limitations: “The inaccuracies of creatinine-based estimates of GFR in children are well known, especially in children with reduced muscle mass (…). Recent studies have addressed the use of other endogenous markers, such as cystatin C, a ubiquitous nonglycosylated cysteine protease inhibitor protein that is produced at a relatively constant rate and is freely filtered by the kidneys (…). This constancy of production is apparently independent of inflammatory conditions, muscle mass, sex, body composition, and age (after 12 mo) (…). Because cystatin C is catabolized and almost completely reabsorbed by renal proximal tubular cells, little is excreted in the urine, so that it cannot be used to measure GFR by standard urinary clearance techniques (…). (…) men and whites may show slightly higher levels than women and blacks, respectively (…).. The latter observations make such a marker difficult to interpret in our multiethnic population (see our attempt at analysis of weight calculation formulas in our multiethnic population [11]).

Future studies need to enroll large cohorts of patients with all degrees of neurodisability to establish normal reference ranges for each severity category.

## 5. Conclusions

Children with neurodisability with mobility restriction requiring physical assistance or powered mobility in most or all settings (GMFCS 4 or 5) have a significantly lower serum creatinine compared to controls with less severe or no neurodisability. Death in severe neurodisability may be associated with lower creatinine levels.

## Figures and Tables

**Table 1:** Personal characteristics, urea, and creatinine levels in children with and without neurodisability.

	Children with neurodisability (n=88)	Age matched controls without neurodisability (n=88)	p-value
Age (mean (SD^a^) in years)	5.8 (3.4)	5.6 (3.4)	0.789
Gender (female)	36	41	0.409
Weight (mean (SD) in years)	19.7 (9.4)	25.2 (16.4)	0.018
Serum urea levels (mean (SD) in mmol/l)	3.7 (1.5)	4.1 (1.2)	0.093
Serum creatinine levels (mean (SD), micromol/l)	32.0 (9.3)	36.5 (9.8)	0.002

^a^ SD=standard deviation

## Data Availability

The data used to support the findings of this study are available from the corresponding author upon request.
